# Catechol-Based Hydrogel for Chemical Information Processing

**DOI:** 10.3390/biomimetics2030011

**Published:** 2017-07-03

**Authors:** Eunkyoung Kim, Zhengchun Liu, Yi Liu, William E. Bentley, Gregory F. Payne

**Affiliations:** 1 Institute for Biosystems and Biotechnology Research, University of Maryland, 5115 Plant Sciences Building, College Park, MD 20742, USA; ekim@umd.edu (E.K.); yliu123@umd.edu (Y.L.); bentley@umd.edu (W.E.B.); 2 Fischell Department of Bioengineering, University of Maryland, College Park, MD 20742, USA; 3 Hunan Key Laboratory for Super Microstructure and Ultrafast Process, School of Physics and Electronics, Central South University, Changsha 410083, China; liuzhengchunseu@126.com (Z.L.)

**Keywords:** catechol, chitosan, hydrogel, information processing, redox-capacitor, electrochemistry

## Abstract

Catechols offer diverse properties and are used in biology to perform various functions that range from adhesion (e.g., mussel proteins) to neurotransmission (e.g., dopamine), and mimicking the capabilities of biological catechols have yielded important new materials (e.g., polydopamine). It is well known that catechols are also redox-active and we have observed that biomimetic catechol-modified chitosan films are redox-active and possess interesting molecular electronic properties. In particular, these films can accept, store and donate electrons, and thus offer redox-capacitor capabilities. We are enlisting these capabilities to bridge communication between biology and electronics. Specifically, we are investigating an interactive redox-probing approach to access redox-based chemical information and convert this information into an electrical modality that facilitates analysis by methods from signal processing. In this review, we describe the broad vision and then cite recent examples in which the catechol–chitosan redox-capacitor can assist in accessing and understanding chemical information. Further, this redox-capacitor can be coupled with synthetic biology to enhance the power of chemical information processing. Potentially, the progress with this biomimetic catechol–chitosan film may even help in understanding how biology uses the redox properties of catechols for redox signaling.

## 1. Introduction

### 1.1. Background: Redox-Active Catecholic Materials

Biology uses materials to perform diverse and important functions, and our understanding of these materials enables biomimetic efforts. For instance, studies of the adhesive properties of mussel proteins identified catecholic residues as critical for both surface binding (adhesion) and protein crosslinking (cohesion), and this knowledge enabled biomimetic materials with broad applications (e.g., polydopamine) [1,2,3,4,5]. Catecholic residues are also redox-active, which means that catechol residues can be switched between oxidized (quinone) and reduced (hydroquinone) states by exchanging electrons with diffusible mediators [6,7,8]. There are several studies to show that the redox activities of catecholic residues can be relevant to some biological functions such as redox buffering [9,10], antioxidant protection [11], metal chelation [12], redox signaling [13] and electron transport [6,14,15]. Although some attempts have been made to study the redox activities of catecholic residues [7,16,17,18], the mechanistic understanding is still lacking because there are few suitable techniques. We are investigating the redox activities of biomimetic catecholic materials and their biological relevance using a recently developed electrochemical reverse engineering method. We believe these redox-active catecholic materials may offer unique technological opportunities for processing chemical information. Thus, our approach may not be truly biomimetic since we are not guided by an understanding of how biology uses melanin’s redox properties to perform functions. We have rather observed intriguing redox properties of biomimetic catecholic materials and we are pursuing technological applications that may or may not be related to their biological function. 

### 1.2. Vision: A New Paradigm for Accessing Chemical Information

Over the last 50 years, advances in information processing transformed our lives by changing the way we access, analyze, and transmit information. Each new device seems to be incrementally cheaper, smaller, faster, more powerful, and easier to use than its predecessor. However, this trend does not extend to instruments that acquire and process chemically-related information. For instance, when we need to access critical chemical information in real time, we still rely on dogs to sniff-out this information. Why have the advances in information processing not been extended to the acquisition and processing of chemically-based information? We believe one issue is that the current paradigm of chemical information is too limited, viewing chemical information through the lens of analytical chemistry and characterizing chemical information in terms of chemical composition and concentration [19]. In essence, this paradigm specifies instrument-intensive approaches (e.g., high performance liquid chromatography–mass spectrometry (HPLC–MS)) to access chemical information and incremental advances are generally accompanied by increasing costs and complexity [20,21,22].

We suggest an alternative paradigm for chemical information processing: one that accesses the power of information processing by searching “chemical space” for global signatures. We envision that this search will be rapid, cheap and convenient, but will lack the granular details of chemical composition and concentration that are targets of conventional analytical chemistry and omics approaches [22]. Thus, we envision a signal processing paradigm that is complementary to (not a replacement for) the current paradigm that focuses on composition and concentration [23,24,25,26].

**Figure 1:**
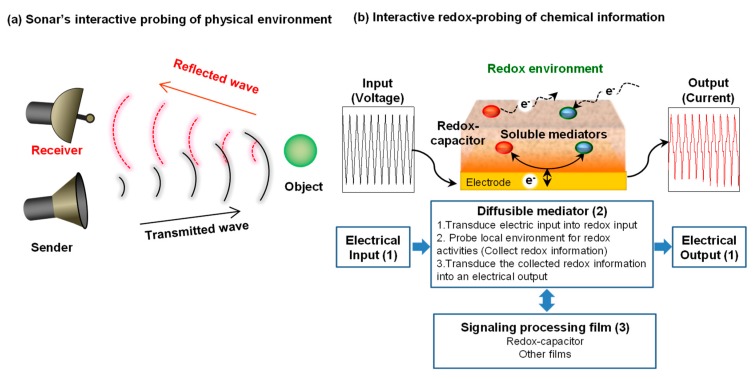
Interactive probing of a local environment. (**a**) Sonar analogy. (**b**) Interactive electrochemical probing consists of: (**1**) complex inputs/outputs to tailor the interactive probing; (**2**) diffusible mediators (electron shuttles) to transmit redox signals that can probe information; and (**3**) signaling processing film to facilitate information processing.

As illustrated in Figure 1a, our signal processing approach is approximately analogous to sonar. Sonar uses a transmitted signal (pressure wave) to propagate through a medium in search of physical information of nearby objects. Interaction with such objects generates a reflected wave that contains information of the objects (e.g., their presence, size, shape and motion).

Figure 1b illustrates our approach to interactively probe for chemical information in a local environment. There are three key features of our interactive electrochemical approach. First is the use of diffusible redox-active mediators (electron shuttles) that serve to transmit redox “signals” that can propagate through the local environment in search of redox-based chemical information. Electron transfer interactions between the mediators and the local environment (i.e., redox reactions) will be detected when the mediator returns to the electrode and engages in electrochemical reactions that serve to transduce the redox information into an electrical output. The second feature illustrated in Figure 1b is use of complex electrical inputs that allows redox-probing to be tailored in search of specific types of information. As will be discussed, the resulting electrical outputs contain information of the mediators’ redox interactions [27,28] and can be analyzed using approaches adapted from signal processing [29,30]. The third feature illustrated in Figure 1b is the use of thin hydrogel film coatings that are used to facilitate signal processing. A catechol-based redox-capacitor is one such film that has proven to be especially useful for processing redox-based chemical information [31,32].

In this review, we will focus on the fabrication and properties of the catechol-based redox-capacitor and we will cite several examples illustrating the value of this capacitor for processing redox-based chemical information.

## 2. Fabrication of Catechol-Based Redox-Capacitor

**Figure 2:**
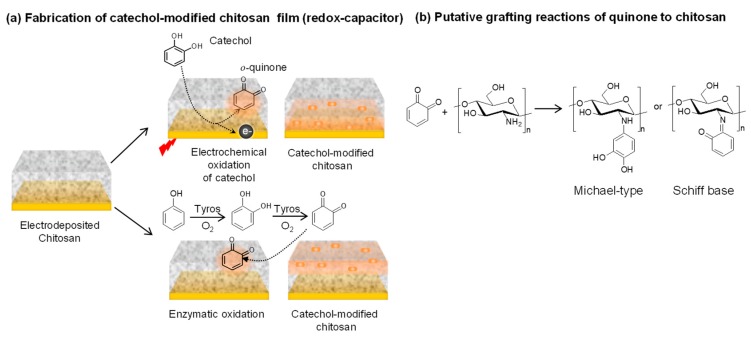
Fabrication of catechol-based redox-capacitor films. (**a**) Catechol is electrochemically oxidized or enzymatically oxidized by tyrosinase (Tyros) and the diffusible oxidation product (*o*-quinone) grafts to the nucleophilic amines of the aminopolysachharide chitosan. (**b**) Quinone grafting likely involves Michael-type and Schiff base adduct formation.

Figure 2a illustrates that the catechol-based redox-capacitor film is fabricated in two steps from catechols and the aminopolysaccharide chitosan [33,34]. The first step is the electrodeposition of a thin chitosan hydrogel film at an electrode surface. This electrodeposition step enlists chitosan’s pH-responsive self-assembling properties [35] and uses cathodic electrolytic reactions to generate the localized high pH that induces chitosan’s neutralization and gel formation [36,37]. In the second step, the chitosan-coated electrode is immersed in a catechol-containing solution and the catechol is then oxidized to generate a reactive *o*-quinone that grafts to chitosan through non-enzymatic reactions. Biologically, enzymes (e.g., tyrosinase) catalyze catechol oxidation [1,38,39] while Figure 2a shows that catechols can also be electrochemically oxidized by biasing the electrode to serve as an anode. Importantly, the chitosan film is a hydrogel that allows diffusion of the catechol reactant and *o*-quinone product. It is also important that the *o*-quinone product is reactive and quickly grafts to chitosan’s primary amines. These quinone grafting reactions are complex and incompletely characterized and likely involve Michael-type adduct and Schiff base chemistries [22,40,41], as suggested in Figure 2b.

## 3. The Catechol–Chitosan Film Can Accept, Store and Donate Electrons

Early studies with melanin suggested that it possesses conducting and semiconductor properties [42,43] and the obvious question was: Are the catechol–chitosan films conducting? Our experimental results indicated that these films are not conducting: electrons do not flow in response to an applied voltage and there does not appear to be direct exchange of electrons with the underlying electrode. This is not surprising given that the films are relatively thick (~1 μm when wet) and the catechols may be too far from the electrode to directly exchange electrons.

**Figure 3:**
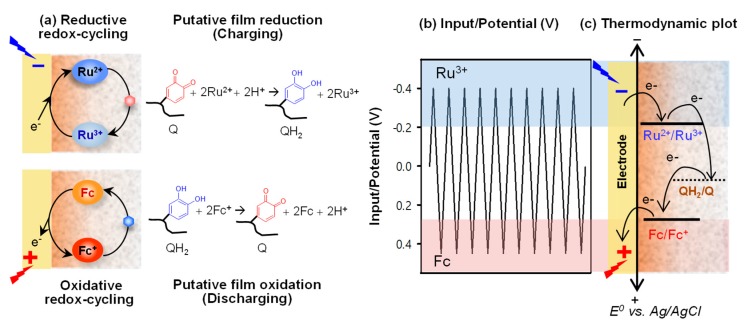
Proposed redox-cycling mechanism of redox-capacitor. (**a**) The reductive redox-cycling reaction of Ru^3+^ can reduce the quinone moieties (charge, QH_2_) and the oxidative redox-cycling reaction of ferrocene dimethanol (Fc) can oxidize the catechol moieties (discharge, Q). (**b**) Electrical input potential. (**c**) Thermodynamics requires electrons to be transferred from more reducing (more negative) potentials to more oxidizing (more positive) potentials.

The next question was: Are the catechol–chitosan films redox-active? Specifically, can the grafted moieties be switched between oxidized states and reduced states? One challenge to assessing the redox switching capabilities of a non-conducting film is what mechanism can be used to transfer electrons from the electrode to the grafted moieties of the film. As illustrated in Figure 3a, we decided to test whether diffusible mediators (i.e., electron shuttles) could be used to engage the catechol–chitosan film in redox-cycling reactions. We found that one mediator, Ru(NH_3_)_6_Cl_3_ (Ru^3+^), could engage in reductive redox-cycling to transfer electrons from the electrode to the film to convert oxidized moieties (Q) to reduced moieties (QH_2_) and thereby charge the film with electrons. A second mediator, ferrocene dimethanol (Fc), could engage in oxidative redox-cycling to transfer electrons from the film to the electrode to convert the film’s QH_2_ to Q and thereby discharge the film.

Importantly, we observed that we could immerse the film-coated electrode into a solution containing both mediators and sequentially engage it in reductive and oxidative redox-cycling reactions if we imposed cyclic voltage inputs as illustrated in Figure 3b. It is also important to note that the “flow” of electrons is constrained by thermodynamics, as illustrated in Figure 3c. The results from these initial studies demonstrated that the catechol–chitosan films are redox-active and can be switched between reduced or oxidized states by exchanging electrons with mediators. 

The observation that the catechol–chitosan films are redox-active but non-conducting leads to two interesting concepts. First, the fact that catechol–chitosan films can accept, store and donate electrons essentially means that they are redox-capacitors. As will be discussed, we use these redox-capacitor properties for information processing in aqueous environments. Second, electrons “flow” through the catechol–chitosan films via distinct electron transfer steps (with intermediates) and not as a “sea” of electrons (as in electron currents in wires). This mechanism is consistent with biological electron transfer reactions (e.g., in the respiratory chain) [44,45] in which electron transfer occurs through distinct stable intermediates. Interestingly, quinones, which are the putative redox-active moieties in our capacitor film, are also important redox-active intermediates in the biological electron transfer chains of both respiration (ubiquinone) [14,15] and photosynthesis (plastoquinone) [13].

## 4. Molecular Electronic Properties of the Catechol–Chitosan Redox-Capacitor

**Figure 4:**
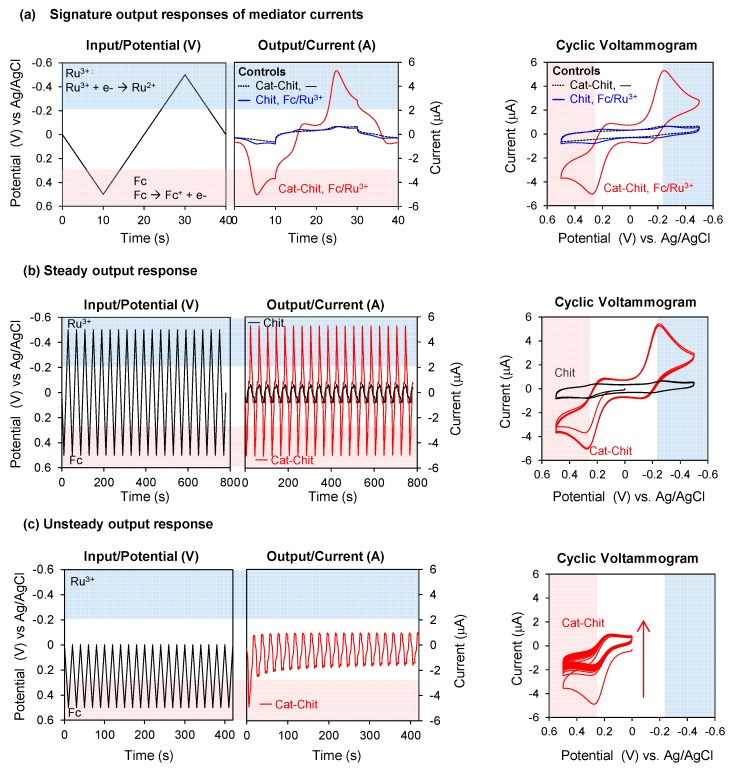
Molecular electronic properties of redox-capacitor. (**a**) Input–output curves and cyclic voltammogram show that the redox-cycling reaction of redox-capacitor with mediators results in output currents that are amplified, partially rectified and gated. (**b**) The output current of mediator is steady over time under unperturbed environment. (**c**) The output current becomes unsteady in the limited range of input potential. Cat-Chit: Catechol-modified chitosan film; Fc: Ferrocene dimethanol. Reproduced from [31] by permission of The Royal Society of Chemistry, 2014.

As a result of its redox activities, the catechol–chitosan redox-capacitor offers interesting molecular electronic properties and we highlight four such properties: amplification, partial rectification, gating, and steady response [31,46,47,48]. The first three properties are illustrated by the results in Figure 4a. In this experiment, the electrode coated with the catechol–chitosan film was immersed in a solution containing both mediators (Fc and Ru^3+^) and a cyclic voltage input was imposed as illustrated by the left plot in Figure 4a. The middle plot of Figure 4a shows the current output response for these studies. The rightmost plot in Figure 4a shows a conventional cyclic voltammogram (CV) which is an alternative representation of the input–output data in which time is not explicitly shown. One control in Figure 4a is incubation of a catechol–chitosan film in the buffer solution without mediators. The output currents for this control show no discernible peaks, which is expected because the catechol–chitosan films are non-conducting. A second control is an electrode coated with a chitosan film (without catechol modification) and immersed in a solution containing both mediators. Results for this control show small output peak currents for Fc and Ru^3+^: these mediators can diffuse through the chitosan film and undergo electron exchange with the underlying electrode. When the electrode coated with the catechol–chitosan film was tested in solutions containing Fc and Ru^3+^, the output peak currents for Fc-oxidation and Ru^3+^-reduction were considerably amplified. Amplification of the mediator currents is consistent with the redox-cycling mechanisms of Figure 3a.

Interestingly, the amplification observed in Figure 4a occurs primarily in one direction for each mediator. The Fc-oxidation current is greatly amplified while the Fc-reduction current is not amplified. The Ru^3+^-reduction current is greatly amplified but the Ru^3+^-oxidation current is not amplified. This partial rectification of the mediator currents is consistent with the thermodynamic plot in Figure 3c, which indicates that Fc can engage in oxidative redox-cycling but not reductive redox-cycling. Similarly, Ru^3+^ can engage in reductive but not oxidative redox-cycling.

To understand the gating property, it is useful to recognize that the currents observed in Figure 4a do not result from direct electron transfer between the film and electrode, but rather are the result of electron transfer between the mediators and electrode. Amplification of these peak currents occurs because the mediators redox-cycle with the film and shuttle electrons between the film and electrode. One requirement for redox-cycling is illustrated by the thermodynamic plot in Figure 3c: a reductive redox-cycling mediator (e.g., Ru^3+^) must have a redox potential (E^0^) that is more reducing than that of the film, and an oxidative redox-cycling mediator (e.g., Fc) must have a E^0^ that is more oxidizing than that of the film. A second requirement for redox-cycling is illustrated by the input voltage curve of Figure 3b: reductive redox-cycling can only occur if the imposed voltage is more reducing than the E^0^ of the reducing mediator, and oxidative redox-cycling can only occur if the imposed voltage is more oxidizing than the E^0^ of the oxidizing mediator (see below for details). In brief, the mediators’ E^0^ plays a critical role in determining if the film can be charged or discharged with electrons, and mediators with differing values can be used to shift the voltage that must be imposed at the electrode to initiate the redox-cycling reactions (i.e., E^0^ of the mediator serves as a gate).

The ability of the catechol–chitosan film to generate steady output responses is illustrated by the experiment in Figure 4b in which an oscillating voltage input was imposed over several hours in the presence of both mediators. The output response (or CV representation) for the electrode coated with the catechol–chitosan film shows that the output currents for both Fc-oxidation and Ru^3+^-reduction were amplified (compared to a control chitosan film) and these amplifications were nearly constant (i.e., steady) over time. From a chemical standpoint, the steady output current responses of Figure 4b indicate that the catechol–chitosan film can be repeatedly switched between oxidized and reduced states. From a signal processing standpoint, steady oscillating inputs and outputs (i.e., sine waves) are integral to the coding and decoding of information.

In contrast to the case of steady input–output, Figure 4c shows an example of unsteady response characteristics. In this unsteady case, the left plot of Figure 4c shows a more limited potential range was imposed (+0.5~0 V) to provide the oxidative voltages required to oxidize Fc but to provide reducing voltages that are insufficient to reduce Ru^3+^. Figure 4c shows the current output response for this unsteady case has no Ru^3+^-reduction peaks and the amplified currents of Fc-oxidation decrease progressively over time. Presumably, this decay in Fc-oxidation currents occurs because the catechol–chitosan film is progressively depleted of electrons during the repetitive Fc-redox-cycling reaction, but this film cannot be replenished with electrons because the imposed voltage is never sufficiently reductive for Ru^3+^-reductive redox-cycling. This unsteady output current response also illustrates the gating function of Ru^3+^: since the imposed voltage remains too oxidative (relative to the E^0^ for Ru^3+^), then reductive redox-cycling mechanism cannot be engaged to recharge the film with electrons. Under this condition, no Ru^3+^-reduction currents are observed and the progressive discharging of the film leads to a progressive decay in Fc-oxidation currents.

The results of Figure 4 illustrate four important molecular electronic properties of the catechol–chitosan redox-capacitor that we use for chemical information processing. One additional feature of this redox-capacitor is illustrated in Table 1. Specifically, the catechol–chitosan redox-capacitor has been observed to accept electrons from a broad range of reductants and donate electrons to various oxidants. This broad ability to exchange electrons with oxidants and reductants indicates that this redox-capacitor has a somewhat generic ability to access redox information (i.e., various different types of mediators can be used). From a chemical standpoint, the broad ability of the catechol–chitosan capacitor to exchange electrons means that it possesses redox catalytic properties and is capable of transferring electrons from reductants to oxidants in response to thermodynamic driving forces. However, we should note that not all redox-active chemicals can exchange electrons with the catechol–chitosan redox-capacitor. Thus, while thermodynamic plots may suggest what reactions are possible, some reactions do not occur within relevant timescales because redox-reactions can have significant kinetic barriers.

## 5. Examples of Chemical Information Processing Using Catechol-Based Redox-Capacitor

As suggested in Figure 1b, we believe that interactive redox-probing can provide access to chemical information, and we are especially focused on accessing chemical information relevant to redox biology. It is well-known that biology uses redox reactions for energy harvesting [56,57] (e.g., electron transfer in the respiratory chain), biosynthesis (e.g., nicotinamide adenine dinucleotide phosphate (NADPH) serves as a diffusible reductant) [10,58,59] and immune response (i.e., the oxidative burst) [60,61,62]. Redox is also emerging as an important signaling modality in biology with the use of diffusible extracellular signaling molecules (e.g., H_2_O_2_) [63,64,65] and redox-based receptor mechanisms (e.g., cysteine-based sulfur switches) [66,67,68]. In addition, redox is recognized as important in biological homeostasis with suggestions that oxidative stress is essentially redox dysregulation. Potentially, probing a local biological environment may reveal information of the redox activities and the redox context [69,70,71].

**Figure 5:**
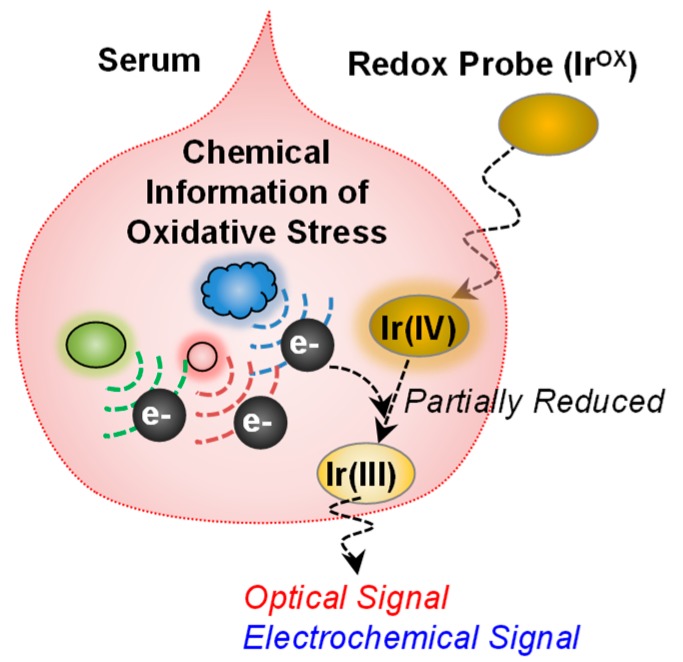
Redox-probing to access chemical information of oxidative stress. The redox mediator (K_2_IrCl_6_, Ir^OX^) can probe chemical information relevant to oxidative stress in blood. The information can be transmitted into optical and electrochemical modalities. Adapted with permission from [55]. Copyright (2017) American Chemical Society.

To illustrate the potential of redox-probing to access complex biological information, we cite recent studies on the development of a blood test for oxidative stress [55]. Specifically, as illustrated in Figure 5, an iridium (Ir) salt was added to serum to serve as an oxidant to detect reducing activities. Subsequent measurements of the amount of Ir reduced could be used to determine an “Ir-reducing capacity”: the lower the Ir-reducing capacity, the greater the oxidative stress. We observed that this measure of oxidative stress could correlate to clinical indicators of schizophrenia and thus this serum assay may aid in the diagnosis and assessment of symptom severity. The important point of this example is that an information processing approach was shown to rapidly access chemical information from serum and this information appears to have considerable clinical utility. Traditional approaches to develop serum tests for schizophrenia generally use panels of analytes, have high costs, and have been unsuccessful in the clinic. The Ir-mediated signal processing approach is essentially a reverse engineering approach in which the Ir mediator is used to probe the serum sample for redox-based information of oxidative stress. This approach does not rely on knowledge of underlying biological mechanisms but probes serum for redox information in a somewhat unbiased way in search of global signatures of relevant information.

### 5.1. Interactive Redox-Probing of Biothiols

Thiols are important moieties in biology because the thiol of glutathione is important for antioxidant protection [72,73,74,75,76] and the thiols of the cysteine residues of proteins are able to serve as redox-responsive crosslinks [74]. Thiols also possess unusual chemical properties and tend to self-assemble onto gold surfaces through gold-thiol interactions rather than transferring their electrons through redox-reactions [77,78]. These chemical properties have made it difficult to detect thiols by electrochemical methods. For instance, the self-assembly of thiols tends to “foul” gold electrodes with insulating monolayer regions (i.e., patches on the gold surface) [79,80,81].

**Figure 6:**
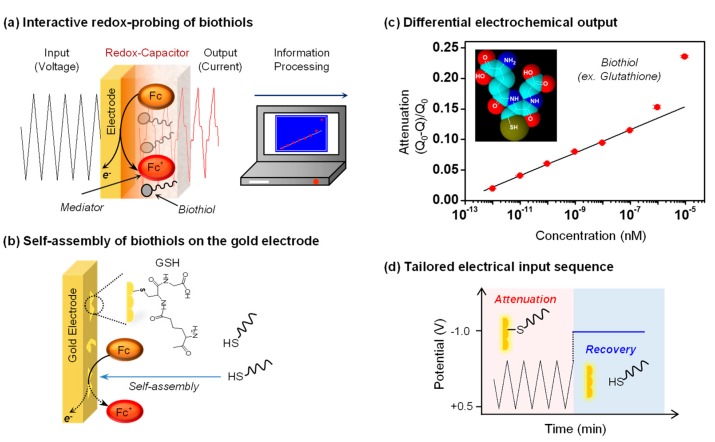
Interactive redox-probing of biothiols. (**a**) The electrochemical information processing approach allows the acquisition of chemical information of biothiols. (**b**) Thiols can self-assemble on gold and potentially attenuate electrochemical signals. (**c**) The quantitative analysis of signal attenuation could be correlated to glutathione (GSH) concentration. Q: Ferrocene dimethanol (Fc)-oxidative charge with biothiol; Q_0_: Fc-oxidative charge without biothiol. (**d**) Tailored electrical input sequence to test the hypothesis that GSH self-assembly on gold attenuates Fc-oxidation. Adapted with permission from [27]. Copyright (2016) American Chemical Society.

We examined whether a redox-probing approach could be used to detect the presence of the biothiol glutathione [27]. In initial studies, we immersed a gold electrode in solutions containing both the Fc and Ru^3+^ mediators and observed that the addition of small amounts of glutathione to this solution resulted in an attenuation of the Fc-oxidation currents. This attenuation is consistent with the self-assembly of the biothiol and a blocking of some of the electrode area to limit Fc-oxidation. When the gold electrode was coated with the catechol–chitosan redox-capacitor film and oscillating voltage inputs were imposed, as illustrated in Figure 6a, the Fc and Ru^3+^ currents were both amplified and the glutathione-induced signal attenuation was more easily detected. Presumably, glutathione can diffuse through the catechol–chitosan and self-assemble onto the underlying electrode as illustrated in Figure 6b. Importantly, Figure 6c shows that quantitative analysis of this signal attenuation could be correlated to glutathione concentration and a linear correlation extended over five orders of magnitude in concentration.

This is an unusual example in the sense that we are using an electrochemical approach to detect the presence glutathione but the method is not based on a redox-reaction between glutathione and either mediator, or between glutathione and the redox-capacitor. Detection is rather believed to result because of the unique chemical capability of thiols to self-assemble onto gold and attenuate the electrode’s ability to oxidize Fc. To provide confirmatory evidence for this mechanism, we tested whether a strong reducing potential that is known to disassemble thiols from gold could be used to reverse the attenuation as illustrated in Figure 6d. We observed that indeed the use of such a reducing potential reversed the attenuated Fc currents and this sequence of attenuation and reversal could be repeated multiple times.

This work illustrates two important points. First, the catechol–chitosan redox-capacitor generates amplified signals that facilitate detection. Second, complex electrical inputs can be imposed to probe for specific information: we used oscillating inputs to generate steady outputs that facilitated quantification of attenuation and we used step changes to reducing potentials to test for biothiol disassembly and a recovery of the Fc-oxidation current. This latter point illustrates that complex electrical input signals can be designed to test specific chemical hypotheses.

### 5.2. Detection of a Redox-Active Bacterial Metabolite

Biology often uses diffusible redox-active metabolites to perform functions: immune cells generate reactive oxygen species to defend against pathogen attack (e.g., the oxidative burst) and H_2_O_2_ is emerging as a diffusible signaling molecule [82,83,84]. Attention has also been focused on other redox-active metabolites such as phenazines, which are among the most-studied redox-active bacterial metabolites [85,86,87]. These metabolites are believed to: (1) allow the producing bacteria to mediate signaling among cells (i.e., quorum sensing) [88]; (2) transfer electrons outside the cell for redox-balancing [89]; and (3) maintain redox homeostasis of multicellular biofilms [90]. One of the phenazines, pyocyanin (PYO), is also a virulence factor for the opportunistic pathogen *Pseudomonas aeruginosa* [85,91]. Importantly, *P. aeruginosa* is emerging as one of the most significant pathogens of nosocomial (hospital acquired) infections, especially for burn patients [92,93,94]. A rapid detection of this pathogen could be integral to successfully identifying and treating infections in this vulnerable patient population.

**Figure 7:**
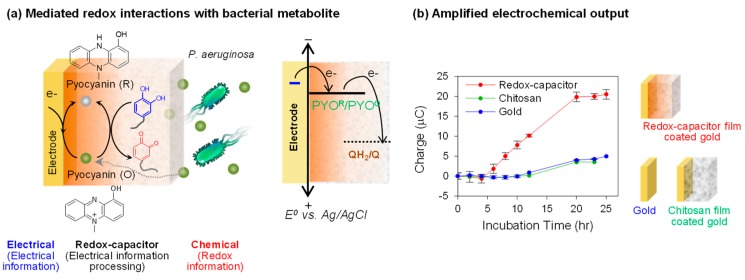
Electrochemical detection of the redox-active bacterial metabolite pyocyanin (PYO). (**a**) Schematic shows that the redox-capacitor can amplify the reduction current of PYO due to the reductive redox-cycling reaction. QH_2_/Q: Catechol(QH_2_) moieties/quinone (Q) moieties of catechol–chitosan film. (**b**) Compared with bare gold and chitosan-coated electrode, the redox-capacitor can sensitively detect the PYO production by bacteria. Adapted with permission from [50]. Copyright (2013) American Chemical Society.

Figure 7a shows that *P. aeruginosa* secretes the redox-active metabolite PYO that can diffuse through the redox-capacitor film [50]. The diffused PYO can be electrochemically reduced at the electrode when the cathodic potential is applied and then the reduced PYO can diffuse back into the film and donate its electrons to the redox-capacitor film. Thus, PYO can undergo reductive redox-cycling with the redox capacitor film and yield amplified output currents that could facilitate detection of *P. aeruginosa*.

To illustrate the sensitive detection of PYO production, we immersed an electrode coated with the catechol–chitosan redox-capacitor into a growing bacterial culture and intermittently performed in situ electrochemical measurement (using a chronocoulometric technique). Figure 7b compares that the charge transfer (a measure of the number of electrons transferred across the electrode) for PYO-reduction against two controls, a bare gold electrode and an electrode coated with an unmodified chitosan film. Both controls show very small charge transfer for PYO-reduction compared to the amplified output generated by the electrode coated by the catechol–chitosan redox-capacitor film.

In summary, the results in Figure 7 demonstrate that the redox-active metabolite (PYO) can undergo redox interactions with the electrode and the capacitor film, and these interactions lead to amplified electrical output signals. In essence, these redox interactions serve to convert chemical information of the bacterial generation of PYO into an electrical output. Potentially, this amplified detection might allow the early detection of infections by the opportunistic pathogen, *P. aeruginosa*. In addition, this measurement may be useful for studying the spatiotemporal dynamics of PYO generation in complex systems (e.g., bacterial biofilms).

### 5.3. A Global Analysis of Redox Context

**Figure 8:**
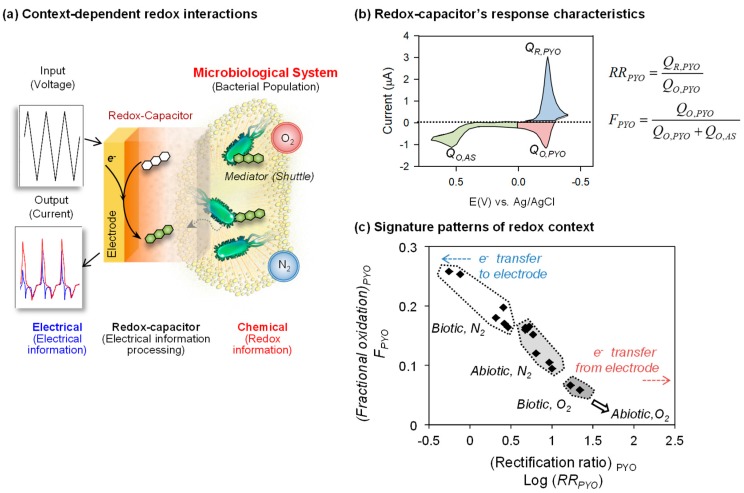
Global analysis of redox context. (**a**) Schematic illustrates that cyclic electrical inputs–outputs are transferred via mediators to the aqueous/biological system, while the catechol–chitosan interface “processes” this information. (**b**) Parameters calculated from cyclic voltammograms that correlate data based on rectification of pyocyanin (PYO) currents and the fraction of total electrochemical oxidation that is attributed to PYO. Q_R,PYO_: PYO-reductive charge; Q_O,PYO_: PYO-oxidative charge; Q_O,AS_: Acetosyrigone (AS)-oxidative charge. (**c**) Two parameters (rectification ratio for pyocyanin (*RR_PYO_*) and fraction of electrochemical oxidation occurring in the pyocyanin region (*F_PYO_*)) show the correlation for the four experimental contexts. Adapted with permission from [95]. Copyright (2013) American Chemical Society.

Figure 1b suggests that redox-probing and signal processing can provide a new paradigm for accessing redox-based chemical information. Our initial effort to measure global redox information is illustrated in Figure 8a in which we immersed an electrode coated with a catechol–chitosan redox-capacitor into a complex bacterial culture and imposed cyclic potential inputs [95]. As noted, these electrical inputs are transduced into redox “transmissions” by the mediators that diffuse through the film into the local environment to probe for redox information (i.e., to assess the redox context). For the example in Figure 8, we added two redox-active biological mediators: the bacterial phenazine pyocyanin that undergoes reductive redox-cycling for film-charging, and the plant phenolic acetosyringone (AS) that can undergo oxidative redox-cycling for film discharging. As an aside, it is useful to note that both molecules are believed to perform signaling functions in biology: PYO for bacterial quorum sensing and AS for a plant innate immune response [96,97,98]. In this example, the catechol–chitosan redox-capacitor films serve to manipulate the redox signals in ways that facilitate interpretation (e.g., the capacitor amplifies and partially rectifies the currents).

To evaluate the signal processing approach, we exposed this capacitor-coated electrode to different redox contexts based on whether the experimental system did or did not have a living population of *Escherichia coli* (biotic or abiotic) or whether there was or was not oxygen present (aerobic or anaerobic). Figure 8b shows a typical CV output response for this experiment and illustrates the signal analysis approach for analyzing the signal. As illustrated in Figure 8b, the CV signal was divided into three regions which were operationally assigned to the specific chemical processes of PYO-reduction, PYO-oxidation and AS-oxidation (note these assignments are important for signal processing but are approximations of the underlying chemistries) [53]. The currents (I) in these regions were integrated with respect to time (t) to determine the charge transfer (Q=∫Idt) in these three regions. These values were then used to generate either a rectification ratio for pyocyanin (*RR_PYO_*) or the fraction of electrochemical oxidation occurring in the pyocyanin region (*F_PYO_*). Using these analytical values, the individual CVs for the four different redox contexts were compared as illustrated in Figure 8c. The correlation plot of Figure 8c shows the ability to discern these four redox contexts (see original paper [95] for details).

In summary, the results in Figure 8 illustrate that redox-probing and signal processing can provide global signatures capable of discerning difference in redox context. Potentially, this analysis could provide a new approach to extract redox-based chemical information from systems that are not well understood and are difficult to probe by conventional methods (e.g., the microbiome [99]).

### 5.4. Coupling Redox-Probing with Synthetic Biology to Access Biochemical Signals

**Figure 9:**
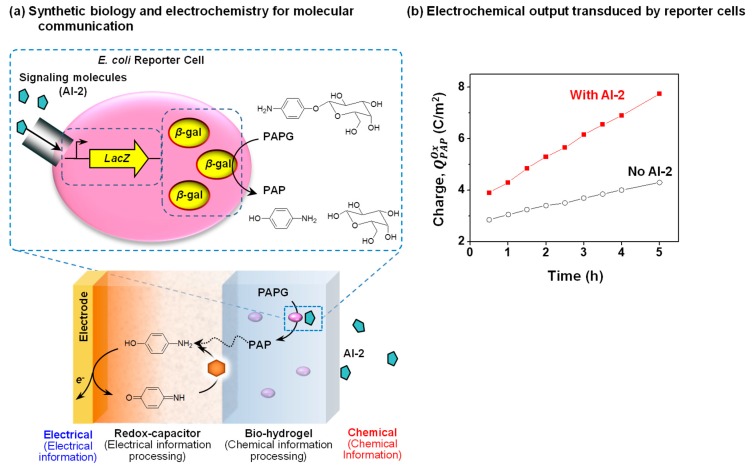
Coupling redox-probing with synthetic biology to access biochemical signals. (**a**) Schematic shows the *Escherichia coli* reporter cell created to detect autoinducer-2 (AI-2) and transduce this information from the redox-inactive substrate *p*-aminophenly β-d-galactopyranoside (PAPG) into a redox-active intermediate that is electrochemically detected. The redox-capacitor converts *p*-aminophenol (PAP) into an amplified electrochemical output. (**b**) The electrochemical output (oxidative charge, *Q^Ox^*) of the dual-film containing *E. coli* reporter cells shows faster increase in the presence of the signaling molecule AI-2 compared with its absence. Reproduced with permission from [54], published by John Wiley and Sons, 2017.

As observed in previous example, some biologically important chemicals are redox-active (e.g., signaling molecules PYO and AS). In these cases, electrochemical methods can be used for direct detection. However, not all molecules are redox-active and in these cases, electrochemistry cannot be directly employed for detection. An emerging approach is to enlist synthetic biology (synbio) to create engineered cells that can recognize a specific chemical and transduce this recognition event into a redox-based signal. For instance, an important bacterial quorum sensing molecule, autoinducer-2 (AI-2), is not redox-active and a synbio reporter cell has been constructed to recognize AI-2 and convert this chemical information into a redox signal that can be electrochemically detected [23,54]. Figure 9a illustrates that this *E. coli* reporter cell transduces the AI-2 molecular input into the expression of the enzyme β-galactosidase (β-gal) that can convert a redox-inactive substrate (*p*-aminophenly β-d-galactopyranoside (PAPG)) into a redox-active product (*p*-aminophenol (PAP)).

Figure 9a also shows that a dual-film system is used to interface these *E. coli* reporter cells adjacent to an electrode. Specifically, these cells are entrapped within a Ca^2+^-alginate bio-hydrogel film that is electroaddressed on top of the catechol–chitosan redox-capacitor film [100,101,102]. The redox-inactive PAPG substrate is purposefully added to the system, and it can diffuse into the dual-film system. When this dual film system is exposed to AI-2, the reporter cells express the β-gal enzyme that converts PAPG into the redox-active PAP product that can diffuse into the redox-capacitor where it can undergo oxidative redox-cycling reactions. Figure 9b shows experimental results demonstrating that the oxidative charge transfer is considerably larger when the dual-film was exposed to AI-2 (compared to the control which the dual-film was not exposed to AI-2).

In summary, the dual-film system serves to process the chemical information of AI-2 into an electrical signal by first using a synbio construct to transduce the AI-2 chemical input into a redox intermediate (PAP), and then using the catechol–chitosan redox-capacitor to convert this redox intermediate into an amplified electrical output. There are two broad features of this work. First, the work demonstrates the coupling of synthetic biology, thin film technology, and electrochemistry to convert chemical information into electrical signals. Second, this coupling is enabled by the use of redox as an intermediate modality that bridges the chemical modality of biology and the electrical modality of devices. Redox can bridge these modalities because it shares features of both the molecular and electrical modalities.

## 6. Conclusions and Future Perspectives

Over the past half-century, microelectronics and information technology transformed the way we process information, but these advances have had a relatively small impact on accessing and understanding the nature of chemical information. A key limitation to interfacing advanced electronic technology with biology is to identify a suitable means to span the chemical modalities of biology and the electrical modality of modern devices. We suggest redox provides a means to span these modalities and propose a new paradigm of interactively probing a local environment for redox-based chemical information in a manner that is analogous to sonar. In this approach, we impose electrical inputs and purposely add redox mediators that can diffuse into the environment and transduce electrical inputs into redox signals (e.g., redox transmission). These mediators probe for redox information in the environment and this information is converted at the electrode into electrical signals that can be “decoded” by signal processing strategies to extract the chemical information. In this approach, thin hydrogel films coated onto an electrode can perform important information processing operations.

We summarize several examples to show that a catechol-based redox-capacitor film can serve to process the redox information generated from various biological systems. We envision the catechol–based redox-capacitor could be useful for processing redox-based chemical information because: (1) it facilitates electron exchange with a broad range of oxidants and reductants, and thus may provide a means to globally sample redox information; (2) it is easily assembled on the electrode by chitosan electrodeposition and electrochemical grafting of catechols; and (3) it possesses unique molecular electronic properties (amplification, partial rectification, gating, switching, and steady response) that serve to process electrochemical information.

We anticipate that this new paradigm for accessing chemical information using a signal processing approach could provide insights on: (1) the oxidative/reductive stresses being exerted on a biological system (e.g., due to inflammation or tumor therapy); (2) the oxidative/reductive actions being taken by a biological system (e.g., oxidative burst or redox signaling); (3) the redox-mediated or regulation process (e.g., for disulfide bond formation/cleavage); and (4) the redox interactions that occur among various chemical components (e.g., between redox-cycling drugs and antioxidants in our diet). Obviously, more tests are needed to validate this proposed signal processing approach and also to understand the utility of the information gained from this interactive redox-probing.

## Figures and Tables

**Table 1:** Tested redox chemicals that can redox-interact with catechol–chitosan films.

Reductants to Donate Electrons to Catechols	Oxidants to Accept Electrons from Catechols
nicotinamide adenine dinucleotide phosphate (NADPH) [49], glutathione (GSH), ascorbic acid [49], pyocyanin (PYO) [50], paraquat [51], Ru(NH_3_)_6_Cl_3_ (Ru^3+^) [47]	O_2_ [49], acetosyringone (AS) [49], clozapine [52], acetaminophen [53], *p*-aminophenol (*p*-AP) [54], K_2_IrCl_6_ (Ir^4+^) [55], ferrocene dimethanol (Fc) [47]
