# Erratum: Catechol-Based Hydrogel for Chemical Information Processing. *Biomimetics* 2017, *2*, 11

**DOI:** 10.3390/biomimetics3020009

**Published:** 2018-05-07

**Authors:** Eunkyoung Kim, Zhengchun Liu, Yi Liu, William E. Bentley, Gregory F. Payne

**Affiliations:** 1 Institute for Biosystems and Biotechnology Research, University of Maryland, 5115 Plant Sciences Building, College Park, MD 20742, USA; ekim@umd.edu (E.K.); yliu123@umd.edu (Y.L.); bentley@umd.edu (W.E.B.); 2 Fischell Department of Bioengineering, University of Maryland, College Park, MD 20742, USA; 3 Hunan Key Laboratory for Super Microstructure and Ultrafast Process, School of Physics and Electronics, Central South University, Changsha 410083, China; liuzhengchunseu@126.com

It was brought to our attention that there were errors in the original publication by Kim et al. [1].

1. One of the authors’ names was misspelled, “Zhenchun” has been corrected to “Zhengchun”.
2. In Reference 102, the author’s names and last names were inadvertently switched,
  “Yi, L.; Eunkyoung, K.; Reza, G.; Gary, W.R.; James, N.C.; William, E.B.; Gregory, F.P. Biofabrication to build the biology–device interface. *Biofabrication* **2010**, *2*, 022002.”
  has been corrected to
  “Liu, Y.; Kim, E.; Ghodssi, R.; Rubloff, G.W.; Culver, J.N.; Bentley, W.E.; Payne, G.F. Biofabrication to build the biology–device interface. *Biofabrication* **2010**, *2*, 022002.”

We apologize for this error.
